# New Frontiers for Selective Biosensing with Biomembrane-Based Organic Transistors

**DOI:** 10.1021/acsnano.0c07053

**Published:** 2020-10-14

**Authors:** Claudia Lubrano, Giovanni Maria Matrone, Gennaro Iaconis, Francesca Santoro

**Affiliations:** † Tissue Electronics, Istituto Italiano di Tecnologia, 80125 Naples, Italy; ‡ Dipartimento di Chimica, Materiali e Produzione Industriale, Università di Napoli Federico II, 80126 Naples, Italy; § Department of Medicine, University of Cambridge, Cambridge CB2 1TN, United Kingdom

## Abstract

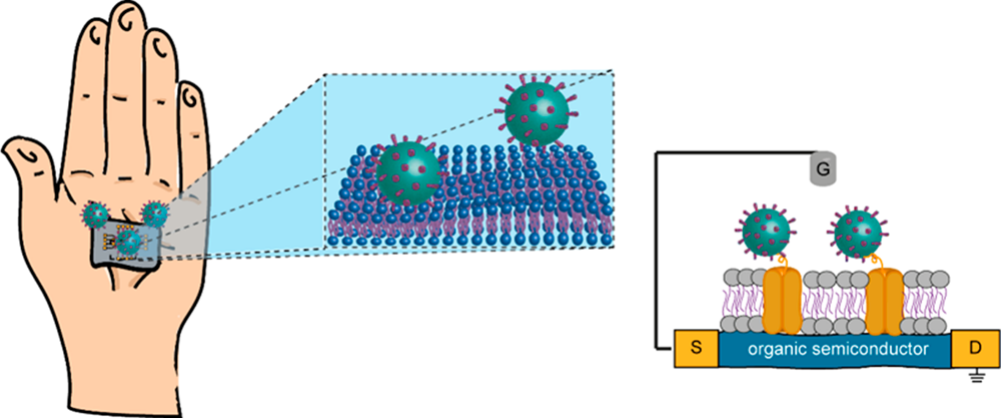

Biosensing plays vital roles in multiple fields, including healthcare monitoring, drug screening, disease diagnosis, and environmental pollution control. In recent years, transistor-based devices have been considered to be valid platforms for fast, low-cost sensing of diverse analytes. Without additional functionalization, however, these devices lack selectivity; several strategies have been developed for the direct immobilization of bioreceptors on the transistor surface to improve detection capabilities. In this scenario, organic transistors have gained attention for their abilities to be coupled to biological systems and to detect biomolecules. In this Perspective, we discuss recent developments in organic-transistor-based biosensors, highlighting how their coupling with artificial membranes provides a strategy to improve sensitivity and selectivity in biosensing applications. Looking at future applications, this class of biosensors represents a breakthrough starting point for implementing multimodal high-throughput screening platforms.

Biosensors are analytical devices that are characterized by a dual configuration: they combine biological elements (*i.e.*, enzyme and receptors) with electronic transduction. As a result, these devices have high selectivity and straightforward processing of the output signals, which can be correlated with the concentrations of target analytes.^1^ Because of their low production costs and high sensing efficiencies, biosensors have applications in a wide variety of fields, especially for the pharmaceutical industry, which demands high-throughput screening platforms to test multiple drugs at a time while preserving high sensitivity and selectivity.^2,3^ As recently experienced during the COVID-19 pandemic outbreak, fast and accurate tests on a large number of samples offer the possibility of decelerating or even preventing the spread of emerging infectious diseases.^4^ Recent events highlight the critical need for portable devices with fast response times (*i.e.*, transistor-based biosensors) as alternatives to conventional biosensing methods (*i.e.*, liquid chromatography, mass spectrometry), ensuring high selectivity and instantaneous measurements.^5^

The use of field-effect transistors (FETs) as biosensors provides economical solutions for efficient transduction platforms of certain analytes that can specifically interact with a transistor-sensitive element (electrode, channel, dielectric layer). Depending on the application, different materials can be employed for the electrode fabrication, usually being classified as metal-, carbon-, or polymer-based.^6^ For instance, metal-based transistors have been used successfully to detect a plethora of bioanalytes, including glucose and neurotransmitters, and were further engineered for immunodetection of biomarkers for early diagnosis of Alzheimer’s disease.^7^ Significant efforts have been devoted to tailoring *ad hoc* surface-functionalizing molecules to manipulate inorganic materials interfaces and to enhance selectivity.^8^ The random orientation of the bioreceptors on the surfaces of FETs might prevent recognition of the target analyte, thus causing artifacts in the output signal. For this reason, recent studies have focused on the development of biomimetic interfaces by means of synthetic cell membranes that are able to preserve the native orientation and conformation of specific biomolecules.^9^

In general, cell membranes act as a barrier, only allowing specific molecules to permeate across the intracellular and extracellular domains. In particular, intercellular trafficking is controlled by transmembrane proteins (TMPs) such as ion channels, which are responsible for the selective passage of cations, small molecules, and metabolites. The opening of these channels is triggered by external stimuli, that is, the specific binding of ligands to their receptors in the cell membrane.^10^ To characterize these ligand–receptor interactions, biomimetic *in vitro* models of cell membranes were established by engineering artificial lipid bilayers on solid supports (supported lipid bilayers, SLBs), such as electrodes and bioelectronic sensors.^11^ The electrical properties of the biosensor can be exploited to monitor the behavior of the SLBs’ insulating double layer. Furthermore, the recombination and activity of TMPs within the SLBs can be detected through capacitive changes at the biosensor–SLB interface.^12^ For instance, Gong and co-workers placed a biomembrane on a carbon-based FET and investigated the role of pore-forming toxins, monitoring the variations in the local charge distribution at the FET surface.^13^ Saem *et al.* employed microstructured electrodes functionalized with lipid bilayers to identify the mechanism of toxic agents on cell membranes and different disruption processes according to the administered chemicals.^14^

Artificial bilayers with embedded proteins can also be exploited to monitor interactions with the corresponding ligands. The material composition employed as the support to the synthetic bilayer plays a critical role: contact between the protruding protein domain and a rigid material surface might cause limited protein mobility or denaturation.^15,16^ In this context, organic conductive polymers are excellent candidates for direct sensing and coupling with SLBs because of their electronic and physical (*i.e.*, swelling, Young’s moduli) properties.^17,18^ For instance, the Daniel group conducted extensive work assembling SLBs on conjugated polymers, such as poly(3,4-ethylenedioxythiophene):poly(styrenesulfonate) (PEDOT:PSS), to achieve selective biosensing through organic electrochemical transistors (OECTs).^19−22^

In this Perspective, we illustrate the advantages of OECTs for biosensing, highlighting opportunities and challenges derived from their coupling with SLBs. We discuss future developments and possible applications of such biomimetic sensors for high-throughput screening of relevant biomolecules and drug testing, as well as their potential role during the COVID-19 pandemic outbreak. These biosensors exemplify a breakthrough in biodetection, promoting a transition from traditional passive sensors toward smart multimodal biosensors.

## Principles of Biosensing in Organic Transistors

**Figure 1:**
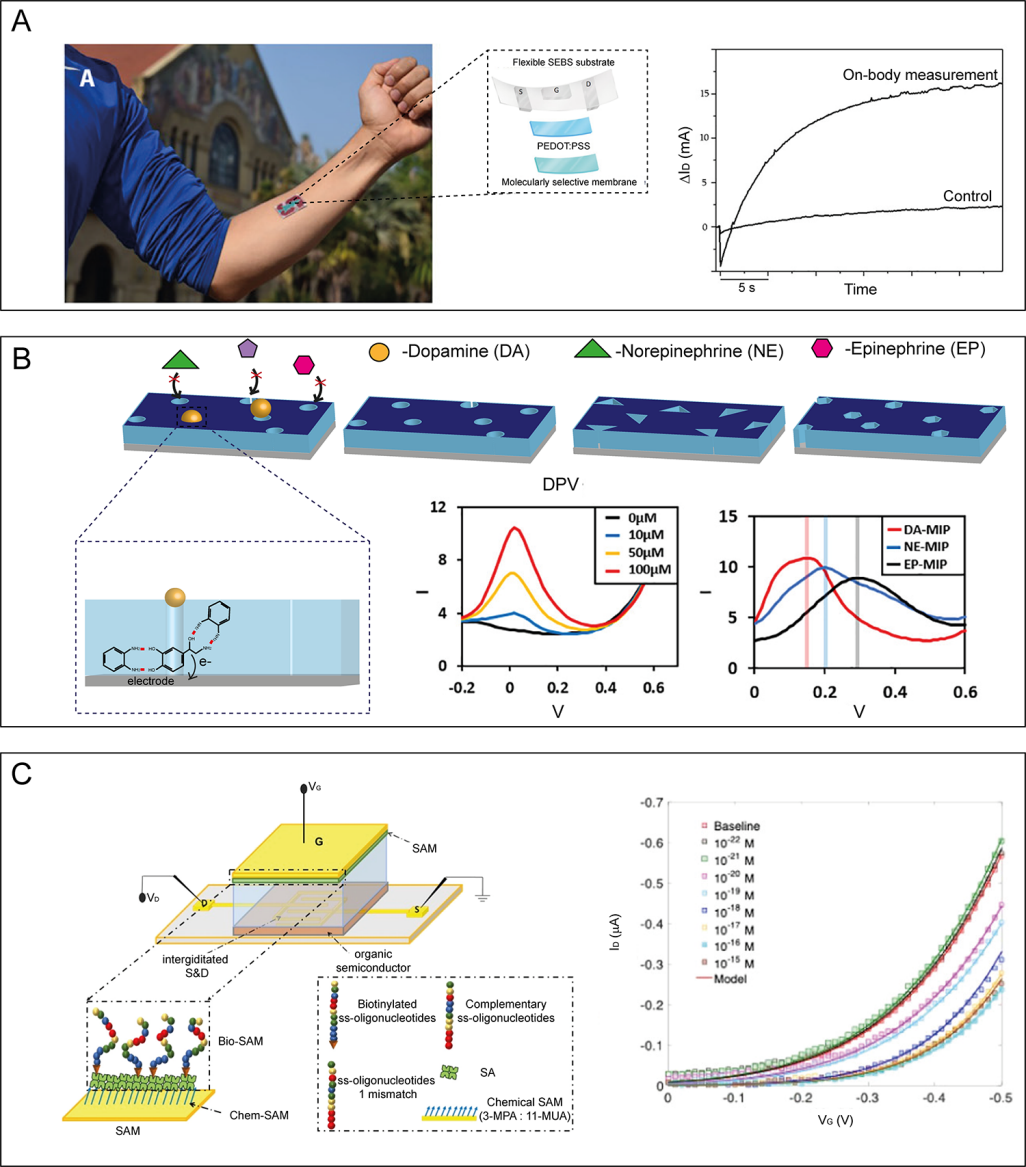
(A) Schematics of the patch-type wearable cortisol sensor. The device was applied on the forearm of a male volunteer, and the real-time responses of both the molecularly selective and control devices were measured after completing physical exercise by mounting electrical contacts to the wearable sensor device (right panel). The cortisol response was recorded using the output measurement, and data were represented as a change of drain current (ΔID). Reprinted and adapted with permission from ref (33). Copyright 2018 American Association for the Advancement of Science. (B) Schematics of the finalization mechanism of molecular imprinted polymers to target different neurotransmitters (DA, NE, EP) and the correspondent differential pulse voltammetry responses at different concentrations. Reprinted and adapted with permission from ref (34). Copyright 2018 Elsevier. (C) Left: Schematic representation of the SiMoT device with the gate surface biofunctionalized with a biotinylated single-strand oligonucleotide. Right: SiMoT transfer *I*–*V* curves measured upon exposure of the gate to standard solutions of target oligonucleotides at the following concentrations: 0.1 zM (black squares), 1 zM (green squares), 10 zM (magenta squares), 102 zM (dark cyan squares), 103 zM (blue squares), and 104 zM (dark yellow squares). Reprinted and adapted from ref (39). Copyright 2020 American Chemical Society.

Organic transistors leverage conjugated polymers as active/sensing channels, featuring the characteristics of coupling mixed electronic and ionic conduction mechanisms. According to their working principles, organic transistors can be divided into three categories: organic field-effect transistors (OFETs), electrolyte-gated OFETs (EGOFETs), and OECTs. Organic field-effect transistors exploit the dielectric layer’s capacitive effect to charge the channel surface, whereas EGOFETs introduce an electrolyte between the gate and the channel. In this case, the application of a bias at the gate drives ions from the electrolyte to the surface of the organic semiconductor, inducing the formation of an electric double layer. In OECTs, the gate voltage causes actual penetration of ions into the bulk of the organic semiconductor, leading to electrochemical doping/dedoping of the channel, thus modulating its transconductance.^23,24^ Organic transistors have been successfully employed for biosensing to detect multiple analytes of biological interest.^25^ In order to improve detection limits and molecular selectivity, different device architectures have been investigated.^26^ Enhanced selectivity can be achieved by modifying the chemical structure of the active materials of OECTs, leading to the detection of electroactive molecules with similar redox potentials.^27,28^ In this respect, electroactive biomolecules can be directly oxidized or reduced on the conjugated polymer electrode, eliciting a characteristic electrochemical response that amplifies the input signal. By exploiting the synergistic effects of the gate and channel for redox signal transduction, a variety of neurotransmitters might be discriminated, combining electrochemical methods with the OECT architecture.^28,29^ Although they enhance the output signal, organic semiconductors might suffer from nonspecific interactions with a variety of biological analytes. Diverse approaches have been developed to improve molecular selectivity of organic transistors through the immobilization of specific bioreceptors on the surface of the device channel. This surface functionalization can be provided by van der Waals interactions or chemical bonds.^30^ As a side effect, however, most organic semiconductors might be affected by conventional chemical processing. Surface treatments, such as oxygen plasma, that do not require aggressive chemical agents represent a valid alternative for introducing functional groups, which could further bind target molecules.^31^ Other species present in the electrolyte may interfere with the mechanism of sensing; however, charged bilayers have been shown to filter these out, enabling selective detection of the target analyte while also increasing sensitivity.^32^ Recently, Parlak and co-workers employed a similar approach to improve the selectivity of OECTs for cortisol biosensing, introducing a synthetic polymeric biological membrane between the gate and the channel of a PEDOT:PSS OECT (Figure 1A).^33^ In the absence of the analyte, this membrane is ion permeable, causing variation of the channel current; in the presence of the analyte, however, the recognition sites are filled and so the membrane is occluded, reducing the current variation. Because receptor grafting may suffer from reproducibility issues and poor surface interactions with the organic sensing layer, molecular imprinted polymers (MIPs) offer a remarkable alternative to increase molecular sensitivity. These polymers are specifically designed to contain molecular cavities whose shapes resemble the molecular structure of the target analyte. During MIP fabrication, the same analytes can be used to template the cavities that work as sensing spots, resulting in an excellent molecular-shape-based specificity that simultaneously enhances the device sensitivity (Figure 1B).^34^

Even after molecular selectivity is addressed, sensitivity remains an issue in organic semiconductors because it is highly affected by the Debye screening effect. Considering common electrolytes and biological fluids, the presence of ions screens the charges of the biomolecules, consequently lowering the detection limit of the device. In this context, although several parameters affect the Debye length (*i.e.*, the decay distance over which the charges are effectively screened), the main contribution corresponds to the ionic strength.^35^ For instance, in physiological buffer such as phosphate-buffered saline (PBS), the Debye length is shorter than the mean length of biomolecules (*i.e.*, antibodies), thus preventing their detection in high-salt electrolytes.^36^ In this case, diluting the electrolyte solution represents a clever approach to overcome the Debye effect; however, this method is not always applicable because specific values of ionic strength could be required to preserve the native conformations of biomolecules. Dilution is also not an option *in vivo*. Alternative approaches include decreasing the distance between the sensing electrode and the analyte of interest or engineering the device architecture with a low-*k* ion-blocking layer for reversible detection of proteins.^37^ Ultimately, the Debye screening effect can be reduced, thus decreasing the dielectric constant of the electrolyte (*i.e.*, adding polymers such as polyethylene glycol, PEG). Detecting small biomolecules with low electric charges (*i.e.*, serotonin, dopamine) represents a difficult challenge unless the charged receptors undergo a conformational change in the same range of the Debye length. Nakatsuka and co-workers proposed the direct immobilization of a bioreceptor on the active layer of the FET to address this issue.^38^ In addition, Macchia and co-workers recently reported a successful strategy to track a biomarker down to the single-molecule level, interfacing a large transistor with a self-assembled densely pack monolayer, which works as a recognition element for both proteins and genomic markers (Figure 1C).^39^

In summary, organic transistors offer excellent molecular selectivity and the processing flexibility to improve and to tune this feature; as a result, they are currently employed as biosensors for a large number of applications. However, there are some technological aspects that have not yet been addressed and, in specific cases, may still limit the application of these architectures in biosensing field. Researchers investigating the molecular design of organic semiconductors are currently trying to balance high mobility, sensibility, and selectivity. Excellent device stability is the key to enable biosensing in complex operating conditions while still offering reproducible results. This latter aspect is usually complicated by the receptor grafting steps, which, even if essential for targeting specific molecules, dramatically reduce device-to-device reproducibility and so their comparability. For example, the immobilization of bioreceptors on the organic semiconductor surface can lead to a random distribution: misalignment prevents the analyte molecules from binding to the antigen epitope or locally alters the molecular interactions with the conductive polymer surface, thus creating artifacts in the output electrical signals.

## Functionalization of Organic Transistors with Biomembranes

As described above, one of the main challenges in biosensing is monitoring key biological signals and interactions without perturbing the system of interest. In a biological environment, bioreceptors are mainly placed on the outer surface of cell membranes or across the lipid double layer. These receptors undergo conformational changes when bound to ligands fitting their recognition site, and in some cases, these changes can cause an exchange of ions between the intracellular and extracellular domains. To investigate certain receptor functions and, ultimately, to advance drug-screening applications through biosensing, the physiological protein conformation and activity should be maintained over time.

**Figure 2:**
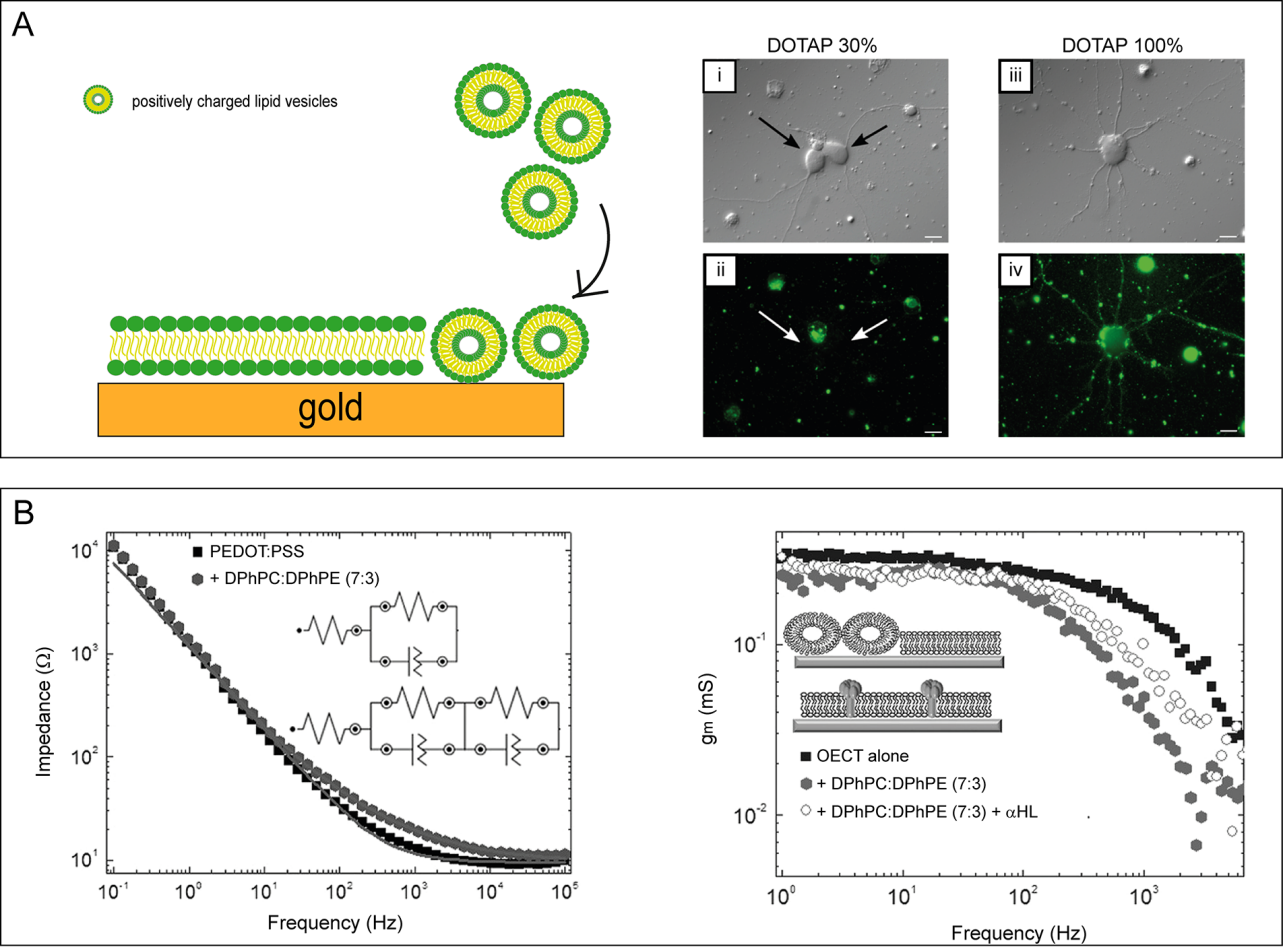
(A) Left: Schematics of positively charged lipid vesicle adsorption and rupture on gold substrate. Right: Bright-field image (i,iii) and fluorescence images (ii,iv) of neurons cultured on a supported lipid bilayer (SLB) on gold (DIV 6). The SLB contains Oregon green 488 1,2-dihexadecanoyl-*sn*-glycero-3-phosphoethanolamine (DHPE) (green fluorescence). The bright fluorescence coming from the cells suggests spontaneous lipid transfer from the bilayer to the cell membrane. However, on 1,2-dioleoyl-3-trimethylammoniumpropane (DOTAP) 30% SLB, only dead cells fluoresce, meaning that live cells are able to resist this transfer process, whereas on DOTAP 100% SLB, both live and dead cells fluoresce. Reprinted and adapted with permission from ref (42). Copyright 2016 American Vacuum Society. (B) Left: Electrochemical impedance analysis of the interaction of lipid vesicles with poly(3,4-ethylenedioxythiophene):polystyrenesulfonate (PEDOT:PSS)-coated sensors. Black squares depict the spectrum of a bare PEDOT:PSS-coated sensor in phosphate-buffered saline (PBS), whereas filled circles indicate the spectrum of the same sensor upon presumed rupture of vesicles. The inset shows the equivalent circuit models in the absence (top) and presence (bottom) of a lipid bilayer. Right: Comparison of transconductance (*g*_m_)–frequency response of PEDOT:PSS organic electrochemical transistor (OECT) alone (black squares) and upon the presumed rupture of vesicles directly on the channel (filled circles). Open circles correspond to the response of the same OECT upon addition of α-hemolysin (α-HL). Cartoon in inset depicts the proposed mixed vesicle/bilayer formed (top) and the insertion of α-HL (bottom). Reprinted and adapted from ref (47). Copyright 2016 Wiley.

**Figure 3:**
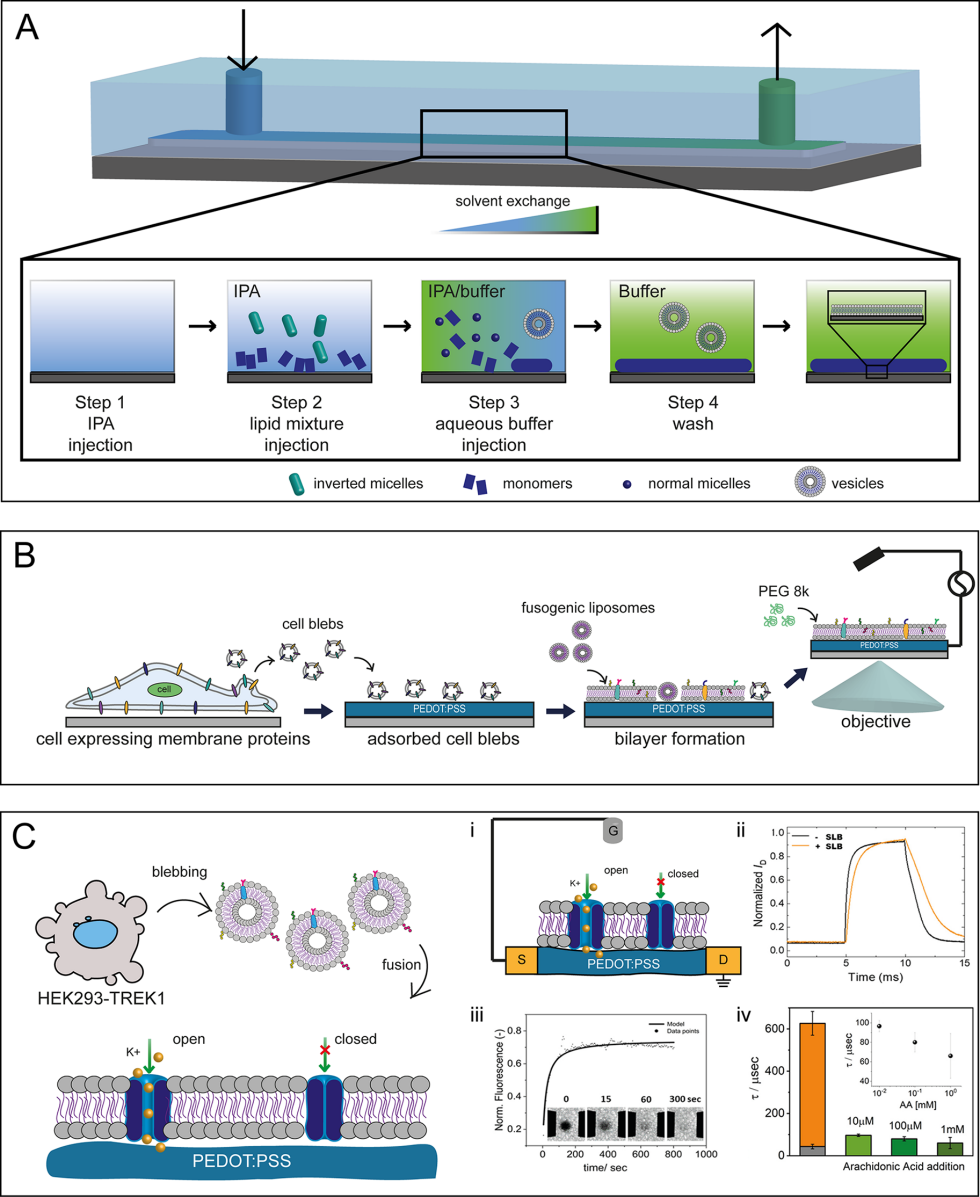
(A) Schematics of the solvent-assisted lipid bilayer formation procedure: (1) introduction of water-miscible organic solvent; (2) addition of lipids dissolved in a water-miscible organic solvent (isopropyl alcohol); (3) exchange of the bulk solution with aqueous buffer; (4) measurement after being washed with aqueous buffer to remove excess lipid molecules, resulting in the formation of a single supported lipid bilayer (SLB) on the underlying solid support. Reprinted and adapted from ref (20). Copyright 2019 American Chemical Society. (B) Schematics of cell bleb rupture and bilayer self-assembly on a poly(3,4-ethylenedioxythiophene):polystyrenesulfonate (PEDOT:PSS) film after the addition of positively charged liposomes and soluble polyethylene glycol 8000 (PEG, 8k). Reprinted and adapted from ref (22). Copyright 2020 American Chemical Society. (C) Left: Schematics of vesicle fusion process on PEDOT:PSS using blebs from the human embryonic kidney (HEK) transfected with the TWIK-related potassium channel (TREK-1, shown in blue). Right: (i) Schematics of an organic electrochemical transistor (OECT) device bearing the HEKTREK- 1 membrane; (ii) temporal response of the drain current; (iii) fluorescence recovery after photobleaching measurements performed simultaneously with the electrical measurements from the same OECT channel (50 μm × 50 μm) shown in (ii); (iv) device response time after application of a square gate bias before and after addition of the TREK-1 activator, AA. The initial device response is shown in gray, and the bilayer with general K^+^ blocker is shown in orange. Green bars denote addition of AA at 10 μm, 100 μm, and 1 mM, as indicated. Inset shows the calibration curve of the device response to different AA concentrations. Reprinted and adapted from ref (21). Copyright 2020 American Chemical Society.

In this context, SLBs are an optimal emulation method for *in vitro* models of biological membranes. These synthetic platforms can be assembled with lipid components of native cell membranes and eventually functionalized with desired proteins. Conventionally, the formation of the artificial double layer is achieved through vesicles fusion (VF),^40^ a well-established procedure based on the spontaneous rupture of lipid vesicles once they reach a critical concentration. However, although this is a straightforward procedure in the case of hydrophilic surfaces (*i.e.*, glass), hydrophobic substrates require pretreatments to adjust the surface tension or specific lipid mixtures to promote surface adhesion (Figure 2A).^41,42^ To overcome such limitations, solvent-assisted lipid bilayer (SALB) formation^43^ might be achieved by rapid solvent exchange from an organic to an aqueous solution, promoting bilayer formation and the possible incorporation of TMPs.^44^ Finally, moving toward fully biological membranes, cell blebbing enables the collection of cell membrane vesicles to form a bilayer with proteins that preserves their native conformations and orientations.^9^ Soft electroactive materials, such as conductive polymers, have recently emerged as ideal supports for these synthetic biomembranes because their high degree of swelling in aqueous conditions^45^ helps to preserve the physiological protein conformation. Among conductive polymers, PEDOT:PSS is used most often in bioelectronics applications because of its biocompatibility, swelling properties, and conduction mechanism transducing ionic-to-electronic current.^46^ However, with the conventional VF method, the correct assembly of SLBs on PEDOT:PSS film has always been a critical challenge, being strictly limited in the choice of lipid vesicles that are able to rupture and to self-assemble spontaneously on its surface (Figure 2B).^47^ Considering possible constraints in the formation of SLBs on conductive polymers, an interesting approach exploited the ability of OECTs to operate in biphasic solvent mixtures. In this configuration, as an alternative to the direct coupling of the SLB with the OECT, a lipid monolayer is placed at the interface between the two immiscible solvents and employed for drug interaction studies: any alteration in the fluidity or packing of the monolayer results in variations in the OECT conductivity.^48^ Recently, implementation of the SALB technique on conductive polymers (Figure 3A) increased the number of membrane models that could be reproduced on conductive polymers, overcoming limitations related to the VF method (*i.e.*, surface tension and electrostatic repulsion).^43^

The possibility of interfacing biomembranes with conductive polymers has been investigated extensively by Daniel and co-workers, who replicated both mammalian and bacterial membranes on PEDOT:PSS films.^20^ They exploited the electrical properties of the conductive polymer to characterize the effect of bacterial toxins and antibiotics on cell membranes: the so-formed models of mammalian and bacterial membranes enable the characterization of single protein functions in a biological pathway of interest, thus providing possible targets for antibiotic therapies.^20^ Alternatively, the implementation of blebs-based bilayers on conductive polymers paves the way toward new biomimetic membrane-functionalized biosensors that display the complexity of native membranes. Liu and co-workers demonstrated that the presence of a hydrated polymeric support not only preserves the mobility and conformation of TMPs but also enables multimodal sensing, combining electrical properties with optical transparency (Figure 3B).^22^ In this issue of *ACS Nano*, Pappa and co-workers describe reconstituting a native human membrane on the PEDOT:PSS channel of an OECT, collecting blebs from cells expressing TREK-1 (a K^+^ ion channel) TMP. The authors then investigated the multimodal read-out properties of the OECT.^21^ Given the optical transparency of PEDOT:PSS, they could estimate the bilayer formation using conventional techniques such as fluorescence recovery after photobleaching, while the simultaneous monitoring of variations in the response time of the OECT provided information about the ion channel activity (Figure 3C). Furthermore, in the presence of a suppressor, the TREK-1 channels inhibited the passage of ions, thus causing increases in the response time of the OECT; the subsequent addition of an activator caused the aperture of the TREK-1 channels to enhance ion flow into the OECT channel and resulted in an almost complete recovery in the response time of the device.

This study represents an important proof-of-concept to characterize TMPs in their native environment, thus identifying possible targets for drug development. Because of the promising results obtained by coupling OECTs with synthetic membranes, recent efforts have been devoted to the engineering of *ad hoc* conductive polymers designed for coupling with SLBs.^46^ Kawan and co-workers proposed a synthesis of an n-type polymer (hence able to work in accumulation mode) modified with lysine side chains, which showed proper surface hydrophilicity to promote vesicle adsorption and fusion.^49^ As discussed above, the electrical and optical properties of the n-type polymer might be exploited to assess the formation of the double layer. Monitoring the charge distribution at the OECT interface enables characterization of a pore-forming protein inserted in the synthetic membrane.

In conclusion, coupling SLBs and OECTs can minimize possible artifacts during biosensing measurements by mimicking and recapitulating biological systems with particular focus on the investigation of protein activity. Researchers have primarily employed SLBs as a platform to mimic cell membranes; however, conventional formation methods have limited their use in organic bioelectronic platforms. The recent advent of the SALB and blebs techniques opened up new frontiers for the replication of different types of biomembranes on organic semiconductors, paving the way for fast and selective biodetection.

## Can Biomembrane-Based Biosensing Be a Valid Solution for COVID-19 Screening?

For the third time in less than two decades, a zoonotic coronavirus infection has crossed animal barriers to infect the human population. Following respiratory syndrome coronavirus (SARS-CoV) in 2002^50^ and Middle East respiratory syndrome coronavirus (MERS-CoV) in 2012,^51^ in December 2019, the World Health Organization was informed of cases of pneumonia by an unknown infectious agent in Wuhan City in the Hubei province of China.^52^ In January 2020, with disclosure of the genomic sequence,^53^ the unknown etiological agent was associated with a novel coronavirus strain, named Severe Acute Respiratory Syndrome Coronavirus 2 (SARS-CoV-2).^54^

Coronaviruses are positive, single-stranded RNA-enveloped viruses with a spherical shape of approximately 125 nm in diameter, coated with club-shaped spike protein, which protrude from the surface, giving the appearance of a solar corona.^55^ Of the four coronavirus genera (α, β, γ, δ), only α and β constitute a threat for humans.^56^ The spike (S) protein of coronaviruses facilitates viral attachment and entry into target cells.^57^ Entry depends on binding of the surface unit S1 of the S protein to a cellular receptor. In addition, entry requires S protein priming by cellular proteases, which entails S protein cleavage at the S1/S2 and the S2′ site and enables fusion of viral and cellular membranes, a process driven by the S2 subunit. SARS-CoV-2 engages the same receptor used by SARS-CoV, angiotensin-converting enzyme 2 (ACE2) as the entry receptor,^58^ and employs the cellular serine protease TMPRSS2 for S protein priming.^59^

Viruses’ mutation rates are much higher than those of their hosts, in the order of a million times higher. Because of this, during the course of infection, a given viral population exists as a genetically different swarm that is capable of rapidly developing resistance to new drugs. Average mutation rates of RNA viruses are estimated to be 100 times higher compared to DNA viruses. A right balance between the integrity of genetic information and the variability of the genome allows a harmonious development of virulence and evolvability.^60–62^ Replication fidelity represents how accurately a genome (both DNA and RNA) is copied in relation to the template strand. Removal of mismatched nucleotides by polymerase-associated 3′-to-5′ exonuclease (proof-reading) activity prevents the arise of mutations within the genome. Thus, the lack of such activity in certain types of viruses results in an increasing number of mutations fixed into the progeny viral genome.

Although the SARS-CoV-2 RNA-dependent RNA polymerases (RdRps) are reported to have proof-reading activity,^63^ the virus nonetheless shows a high degree of genomic variability. Moreover, numbers of infected cases, deaths, and mortality rates related to COVID-19 vary from country to country.^64^ SARS-CoV-2 has rapidly spread around the world compared with SARS-CoV and MERS-CoV. Although the estimated fatality rate in the confirmed cases is 6.6% in SARS-CoV-2, which is lower than that of SARS-CoV and MERS-CoV at 9.6 and 34.3%, respectively, there is an urgent need for its effective treatment based on antivirals and vaccines that reduce the mortality and morbidity rates of COVID-19.

There are principally two types of tests available for COVID-19: viral tests and antibody tests. Viral tests are direct tests that are designed to detect the virus and, therefore, to reflect current infection. In contrast, antibody tests are indirect tests, as they do not detect the virus but rather ascertain established seroconversion to previous infection or early seroconversion to ongoing infection. Determining whether an individual is currently infected with SARS-CoV-2, until recently, required reverse transcription polymerase chain reaction (RT-PCR) testing. However, understanding whether the RT-PCR test results are interpreted as quantitative, qualitative, or semiquantitative is important. Results for SARS-CoV-2 testing are generally reported qualitatively as positive or negative, even though viral load may provide both clinically and epidemiologically important information. In addition, RT-PCR diagnosis of COVID-19 has limitations. Detecting SARS-CoV-2 from pharyngeal swabs requires high-quality specimens that contain a sufficient amount of intact viral RNA. However, SARS-CoV-2 loads in the respiratory tract have been shown to vary considerably.^65^ This variability has not only led to high false-negative rates, with probable cases remaining negative after multiple swabs, but is also further exposing healthcare workers to risk of infection. Moreover, processing COVID-19 samples requires specialized biocontainment laboratories operated by highly trained technicians, which are usually only found within medium-to-large hospital facilities.

Immunoassays show some distinct advantages over RT-PCR. Antigens and antibodies are considerably more stable than RNA, which makes them less susceptible to spoiling during transport and storage, therefore reducing the chance of false-negative results. Testing accuracy is also improved by the fact that antigens and antibodies are more uniformly available in sputum and blood samples. However, despite the ability to detect past infections, antibodies are only able to provide limited information. If the test is administered too soon after infection, there might not yet be detectable antibodies. In addition, antibody tests require some knowledge of the proteins that form the viral coat—specifically, those proteins that trigger the immune system. Those sections of the viral protein coat must then be produced in the laboratory, using cell lines, for inclusion in an immunoassay (*e.g.*, enzyme-linked immunosorbent assay) that detects whether antibodies are present. This development takes time.

The current pandemic has demanded rapid upscaling of *in vitro* diagnostic assays to enable mass screening and testing of high-risk groups and simultaneous compilation of robust data on past SARS-CoV-2 exposure at both individual and population levels. To meet the greatly increased demand in testing, accelerated development of both molecular and serological assays across a plethora of platforms is underway.

**Figure 4:**
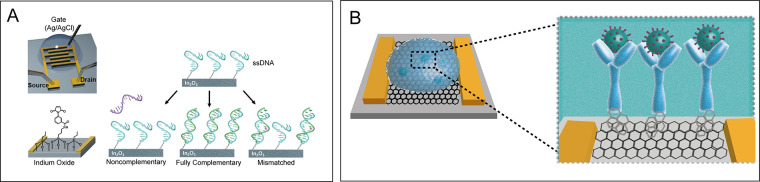
(A) Field-effect transistors (FETs) for detection of oligonucleotides. FETs functionalized with ssDNA were exposed to noncomplementary, fully complementary, or mismatched sequences. Reprinted and adapted from ref (67). Copyright 2020 American Chemical Society. (B) Schematics of COVID-19 FET sensor: the graphene sensing material is conjugated with the SARS-CoV-2 spike antibody, enabling the virus detection. Reprinted and adapted from ref (5). Copyright 2020 American Chemical Society.

Electronic biosensors (*i.e.*, FETs and OECTs) can play important roles in preventing the spread of infectious diseases due to their fast responses and low detection limits. Once the structure of the virus has been decoded, the immobilization of specific antibodies on the FET surface enables targeting proteins placed on the outer shell of the virus, thus enabling the detection of SARS-CoV-2 in clinical samples.^5^ Recently, Funari and co-workers provided an alternative strategy based on surface plasmon resonance rather than on electrical sensing, accomplishing fast and accurate detection of SARS-CoV-2 antibodies in diluted human plasma samples.^66^ In addition, due to the possibility of immobilizing single-stranded oligonucleotides directly on the surface of FETs (Figure 4),^67^ these transistor-based biosensors are able to provide insights about possible mutations in the genetic makeup of the virus, thus providing essential information for the development of new therapies. Ultimately, taking into account recent progress in the coupling of biomembranes with organic transistors, it is realistic to envision a forefront role for this platform in the fight against COVID-19. Once the triggering mechanism of the virus is known, the biomembrane mask of the sensor may be an optimal platform to act as a fake host for the virus.^68^ For instance, the ACE2 receptor can be embedded within the artificial membrane placed on the OECT such that the specific recognition with the S protein of the virus induces the binding and possible fusion of the virus membrane on the device. This binding will result in conductivity changes in the OECT channel, enabling fast and accurate detection of the SARS-CoV-2 virus. Moreover, specific drugs that are potential candidates for therapeutic applications (*e.g.*, calcium channel blockers)^69^ might be further investigated through the engineering of dedicated biomembrane-based organic platforms.

This pandemic further confirmed the efficiency of transistor-based sensors for selective, highly sensitive detection of biomolecules; however, we can estimate from the reported data^21^ that the additional functionalization with biomembranes might provide a significant upgrade to organic-based sensors because these biomimetic platforms can provide not only fast and accurate detection but also potentially a therapeutic treatment, as well.

## Conclusions and Future Perspectives

The direct assembly of synthetic biomembranes on OECTs is expected to revolutionize the field of biosensing by enabling direct detection of biomolecules in their native environments or mimics thereof. Combining SLBs and OECTs solves several issues of pre-existing platforms, including the mismatch between biomolecules and traditional inorganic sensors and the denaturation of TMPs. These biomembrane-based devices are paving the way toward the generation of new classes of biosensors that would combine the advantages of OECTs and SLBs. The possibility of massive parallelization of OECT devices could lead to high-throughput platforms for drug-screening applications and decreasing time and costs of drug testing, thus significantly affecting the pharmaceutical field. In addition, replicating biological systems in their native states offers the chance to study and to characterize biological mechanisms that have been overlooked thus far because of their complexity. This latter aspect may be particularly relevant for neurodegenerative diseases that are still extremely challenging to explore: identifying proteins/receptors that are involved in the early stages of the disease could provide insight into new targets for possible therapeutic treatments. Ultimately, looking at the current COVID-19 outbreak, we envision a future, wearable configuration of such biomimetic sensors that will be able to detect and to inactivate the virus at the same time. From our perspective, it is evident that the pandemic highlights the importance of massive testing and fast screening; however, the most important take home message revealed from this outbreak is the urgent need for faster transition from laboratory research to commercial prototypes for daily life applications.
